# Association between exposure to technological advances in the workplace and work engagement: a prospective cohort study

**DOI:** 10.1093/joccuh/uiae003

**Published:** 2024-01-19

**Authors:** Nuri Purwito Adi, Tomohisa Nagata, Kiminori Odagami, Masako Nagata, Koji Mori

**Affiliations:** Department of Occupational Health Practice and Management, Institute of Industrial Ecological Sciences, University of Occupational and Environmental Health, Kitakyushu 807-8555, Japan; Department of Community Medicine, Faculty of Medicine, Universitas Indonesia, Jakarta 10230, Indonesia; Department of Occupational Health Practice and Management, Institute of Industrial Ecological Sciences, University of Occupational and Environmental Health, Kitakyushu 807-8555, Japan; Department of Occupational Health Practice and Management, Institute of Industrial Ecological Sciences, University of Occupational and Environmental Health, Kitakyushu 807-8555, Japan; Department of Occupational Medicine, School of Medicine, University of Occupational and Environmental Health, Kitakyushu 807-8555, Japan; Department of Occupational Health Practice and Management, Institute of Industrial Ecological Sciences, University of Occupational and Environmental Health, Kitakyushu 807-8555, Japan

**Keywords:** automation, job resources, technology, work performance, information, training

## Abstract

**Objectives:** The study objective was to measure the association between exposure to technological advances and work engagement, adjusting for personal and workplace factors.

**Methods:** We conducted a nationwide online longitudinal survey study in Japan. The sample was stratified to represent Japanese workforce conditions. Work engagement was measured using the Japanese version of the Utrecht Work Engagement Scale-9. Exposure to technological advances was measured using a single question with Likert scale responses. Industry characteristics that were more/less likely to be replaced by automation were also measured. Linear regression was used for statistical analysis.

**Results:** There were 16 629 participants. We found that exposure to technological advances was associated with work engagement after adjustment for age, sex, education, income, and industry characteristics. We observed a significant interaction between age and exposure to technological advances (coefficient 0.891, *P* < .001), and conducted an age-stratified linear regression analysis. The significant association between age and exposure to technological advances reduced as age increased, and disappeared after adjustment for baseline work engagement.

**Conclusions:** Longitudinal observations showed that exposure to technological advances was not significantly associated with work engagement.

## Teaser

**Key points:** **What is already known on this topic**

1. Exposure to technological advances likely contributes to negative outcomes such as job insecurity, poor health conditions, and work-related injury or disease.

**What this study adds**

1. Longitudinal observations showed that exposure to technological advances was not significantly associated with high work engagement.
2. Age moderated the relation between exposure to technological advances and high work engagement; younger workers had higher work engagement.

**How this study might affect research, practice, or policy**

1. Exposure to technological advances such as artificial intelligence should be carefully implemented over a longer period, as such exposure does not seem to relate positively to work engagement. Other resources are likely to be needed to help employees exposed to technological advances maintain their work engagement.

## 1. Introduction

Rapid advances in technology have increased understanding of physical processes and simplified many aspects of life.^1^ A 2019 report showed that the implementation of artificial intelligence (AI) increased by 270% across all industries within 4 years, and owing to the digital transformation of health care, 38% of health care providers rely on computer-assisted diagnostics.^2^ The development of technological advances has also occurred in Japan, which was the first country to demonstrate that it is possible to expand through the use of innovation even as the population declines. Japan is rapidly moving toward the creation of a “Society 5.0,” with the assistance of products related to technological advances such as industrial robots, digital farming, new drug discoveries, and the use of AI in regenerative medicine.^3^

Lee et al^4^ have stated that workers exposed to more technological advances will likely experience greater job insecurity. Other studies have identified potential job losses in occupations susceptible to automation,^5^^-^^7^ and increases in mortality,^8^ poor health conditions,^9^ and work-related injury or disease.^10^ Technological advances also change the characteristics and demands of work by leading to greater work complexity, more mental work tasks, workload changes, and work role expansion. However, technological advances may also have positive effects by reducing manual work and creating more new job opportunities.^11^ The World Economic Forum has estimated that by 2025, although 85 million jobs will be displaced by automation, 97 million new jobs will be created.^12^ These changes will probably depend on the extent to which technological advances affect different job tasks and job positions.

A previous study investigated how acceptance of technology affected work engagement before the COVID-19 pandemic.^13^ The study focused on workers whose work activity had recently changed following the introduction of new technology, and examined worker acceptance of new technology. However, the conditions of exposure to technological advances in real workplace settings may differ; not every worker is exposed to the same extent and duration to newly installed technology. Furthermore, the COVID-19 pandemic may have affected the conditions and longitudinal effects of such exposure.

Workers’ exposure to technological advances increased during the COVID-19 pandemic. Preventive measures such as physical distancing required industries and workers to adapt to new habits like working from home. Workers had to learn new modalities to enable remote working support and install new technologies. The pandemic may also have had psychological effects on workers,^14^ which in turn may have affected worker performance.

In light of pandemic-related changes, there is a need to measure technological advances among workers in real workplace settings using longitudinal data. It is also important to consider the effect of work engagement as one aspect of workers’ psychological performance profile. Such investigation is important to justify the implementation of technological advances, adjusted to industry characteristics, in countries such as Japan, which have a high commitment to technological advances.

As we had defined work engagement as our dependent variable, we used the job demands–resources model (JD-R) as the framework for this study. According to the JD-R model, job resources affect work engagement via motivational processes. Job resources were defined as aspects of the job that are functional in achieving work goals and reducing job demands and the associated physiological and psychological costs.^15^ Our hypothesis was that exposure to technological advances would function as a job resource and would be associated with high work engagement, following Beer and Mulder,^11^ who concluded that increased autonomy and reduced manual work were positive effects of exposure to technology. As there is evidence for a possible interaction between age and exposure to technological advances, in that greater age tends to be associated with less exposure to technological advances,^16^ we investigated the interaction between age and exposure to technological advances.

## 2. Methods

### 2.1 Study design and participants

This was a prospective cohort study conducted in Japan. The study was part of a nationwide online survey targeted to workers, and covered all regions of Japan. The study protocol has been published previously.^17^ The 27 693 participants registered in the initial survey in the first year were screened to produce 16 629 participants who responded in the second-year data collection and who had complete data from the previous year. Details of the comparison between respondents and nonrespondents are shown in Table 1. Stratification sampling was carried out to represent the conditions of the Japanese workforce.^18^ Data were collected in February-March 2022 for the first year and February-March 2023 for the second year. Data collection was supported by a private survey company that is officially registered in Japan and has 2.2 million registered members. Only individuals registered with the company could respond to the survey. The study was approved by the ethics committee of the University of Occupational and Environmental Health, Japan (R3-076) and complies with the Checklist for Reporting Results of Internet E-Surveys (CHERRIES).^19^

**Table 1:** Demographic data of the respondents.

**Variables**	**Exposure to technological advances** ^*^							**Total respondents**	**Total nonrespondents**
	**1**	**2**	**3**	**4**	**5**	**6**	**7**		
***n***	1685	1841	2024	4383	3740	1982	974	16 629	11 064
**Sex, *n* (%)**									
**Male**	815 (48.4)	967 (52.5)	1131 (55.9)	2400 (54.8)	2261 (60.5)	1283 (64.7)	678 (69.6)	9535 (57.3)	5667 (51.2)
**Female**	870 (51.6)	874 (47.5)	893 (44.1)	1983 (45.2)	1479 (39.5)	699 (35.3)	296 (30.4)	7094 (42.7)	5397 (48.8 )
**Age, mean (SD)**	50.9 (12.7)	50.6 (13.2)	47.5 (13.4)	46.4 (12.5)	46.3 (13.1)	46.9 (13.2)	46.3 (12.3)	47.5 (13.1)	42.9 (13.5)
**Education level, *n* (%)**									
**Low**	607 (36)	517 (28.1)	503 (24.9)	1263 (28.8)	771 (20.6)	342 (17.3)	198 (20.3)	4201 (25.3)	2815 (25.4)
**Medium**	491 (29.1)	483 (26.2)	460 (22.7)	1018 (23.2)	735 (19.7)	339 (17.1)	154 (15.8)	3680 (22.1)	2605 (23.5)
**High**	587 (34.9)	841 (45.7)	1061 (52.4)	2102 (48)	2234 (59.7)	1301 (65.6)	622 (63.9)	8748 (52.6 )	5644 (51.0)
**Income category, *n* (%)**									
**<4 million yen**	596 (35.4)	539 (29.3)	525 (25.9)	1190 (27.2)	819 (21.9)	351 (17.7)	196 (20.1)	4216 (25.4)	2710 (24.5)
**4-5.99 million yen**	453 (26.9)	486 (26.4)	520 (25.7)	1113 (25.4)	864 (23.1)	442 (22.3)	209 (21.5)	4087 (24.6)	2832 (25.6)
**6-7.99 million yen**	282 (16.7)	349 (19.0)	414 (20.5)	879 (20.1)	761 (20.3)	417 (21.0)	186 (19.1)	3288 (19.8)	2190 (19.8)
**8-9.99 million yen**	189 (11.2)	243 (13.2)	253 (12.5)	582 (13.3)	597 (16.0)	320 (16.1)	133 (13.7)	2317 (13.9)	1497 (13.5)
**10-12 million yen**	62 (3.7)	89 (4.8)	126 (6.2)	261 (6)	276 (7.4)	177 (8.9)	91 (9.3)	1082 (6.5)	789 (7.1)
**>12 million yen**	103 (6.1)	135 (7.3)	186 (9.2)	358 (8.2)	423 (11.3)	275 (13.9)	159 (16.3)	1639 (9.9 )	1046 (9.5)
**Industry characteristics, *n* (%)**									
**More likely replaced with automation**	383 (22.7)	435 (23.6)	532 (26.3)	1216 (27.7)	1134 (30.3)	668 (33.7)	405 (41.6)	4773 (28.7)	3100 (28 )
**Less likely replaced with automation**	1302 (77.3)	1406 (76.4)	1492 (73.7)	3167 (72.3)	2606 (69.7)	1314 (66.3)	569 (58.4)	11 856 (71.3)	7964 (72 )
**Work engagement score year 1, mean (SD)**	21.2 (14.4)	23.2 (12.1)	22.1 (11.2)	20.6 (12.1)	23.1 (11.2)	23.5 (11.9)	23.2 (14.2)	22.2 (12.2)	22.3 (12.4)
**Work engagement score year 2, mean (SD)**	22.8 (12.6)	24.1 (10.5)	23.5 (9.8)	22.4 (10.4)	23.8 (9.9)	24.4 (10.4)	24.5 (12.6)	23.4 (10.7)	—

^*^Degree of exposure, 1 = not effected - 7 = greatly affected

### 2.2 Work engagement

Work engagement was examined using the 9-item Japanese version of the Utrecht Work Engagement Scale (UWES-9).^20^ Work engagement was measured twice, at baseline and 1 year after the first collection of data on exposure to technological advances. Work engagement at baseline was used for model adjustment. Responses to the 9 UWES-9 questions are on a Likert scale ranging from 0 (never) to 6 (always). Data were presented numerically using total scores; the minimum possible score was 0 and the maximum possible score was 54.

### 2.3 Exposure to technological advances

Exposure to technological advances was measured using a single question: “To what extent do you think your work will be affected by technological advances such as AI and digital transformation?” Participants were asked to respond on a Likert scale ranging from 1 to 7; a score of 1 represented not affected at all and a score of 7 represented greatly affected by technological advances.

### 2.4 Covariates

The covariates were age, sex, education, income, and industry characteristics. Age was expressed numerically based on the data from the online questionnaire. Sex was categorized as male or female. Education level was determined according to the last school that participants graduated from, and categorized as low (junior high school and high school), medium (vocational school and junior college/technical college), and high (university and graduate school). Income was categorized according to 6 categories of annual income: less than 4 million yen, 4.0-5.99 million yen, 6.0-7.99 million yen, 8.0-9.99 million yen, 10.0-12.0 million yen, and more than 12.0 million yen. Industry characteristics were measured according to the Japan Standard Industrial Classification^21^ and categorized into 2 groups based on the likelihood that jobs would be automated.^22^ Information and communication; wholesale and retail trades; finance and insurance; real estate, goods, rental, and leasing; and education and learning support were categorized as industries most likely to have their jobs automated. Manufacturing; transport and postal services; agriculture and forestry; fishery; mining and quarrying stone and gravel; construction; electric; gas; heat and water supplies; scientific research; professional and technical services; accommodation and eating and drinking services; living-related, personal, and amusement services; medical, health care, and welfare; compound services; public services; and unlabeled services were categorized as industries less likely to have their jobs automated.

### 2.5 Statistical analysis

We examined the distribution of data for covariates, exposure to technological advances, and work engagement. Linear regression analysis was performed to examine the association between exposure to technological advances and work engagement. Model 1 was generated with adjustment for age, sex, education, and income. Model 2 was generated from Model 1 with additional adjustment for industry characteristics. Model 3 was generated from Model 2, with additional adjustment for the interaction between age and exposure to technological advances. The Model 3 interaction results were used to inform further analysis to examine the role of age. Model 4 was generated from Model 2 with additional adjustment for work engagement score at baseline. All statistical analyses were performed using SPSS, Faculty Packs version 29 (IBM, Armonk, NY, USA) and Stata statistical software (Release 17; StataCorp LLC, College Station, TX, USA).

## 3. Results

Table 1 shows the demographic data of the participants. There were 16 629 participants and most (71.3%) did not work in industries that were likely to be automated. The mean (SD) work engagement score in year 1 (baseline) (22.2 [12.2]) was slightly lower than that in year 2 (23.4 [10.7]). The mean work engagement scores for both years were in the 50th percentile, indicating moderate work engagement. Compared with respondents, nonrespondents were more likely to be younger and female.

Table 2 shows the results of the simple linear regression analysis of each variable, including exposure to technological advances and work engagement in year 2. Exposure to technological advances was associated with high work engagement in year 2.

Table 3 shows the results of the multiple linear regression analysis. We found that exposure to technological advances was associated with work engagement in year 2 even after adjustment for age, sex, education, and income (Model 1), and after additional adjustment for industry characteristics (Model 2). We also observed that there was a statistically significant negative coefficient for the interaction between age and exposure to technological advances (Model 3). The standardized coefficient of the association between exposure to technological advances and work engagement was 0.138 in this model. A multicollinearity test of the models indicated low multicollinearity (the variance inflation factor was less than 10) except for Model 3, which contained a composite variable. However, in Model 4, the association between exposure to technological advances and work engagement in year 2 disappeared following adjustment for work engagement in year 1.

Table 4 shows the results of the multiple linear regression analysis stratified by age group (20-39 years, 40-59 years, and 60 years or above). Exposure to technological advances was significantly associated with work engagement in year 2 in all age groups, but the regression coefficient tended to decrease as age increased.

**Table 2:** Correlation coefficient between variables including work engagement year 2.

**Variable**	**Exposure to technological advances**	**Age**	**Education level**	**Income category**	**Work engagement year 1**	**Work engagement year 2**
**Exposure to technological advances**	1					
**Age**	−0.111	1				
**Education level**	0.160	−0.094	1			
**Income category**	0.144	0.000	0.238	1		
**Work engagement year 1**	0.040	0.228	0.053	0.105	1	
**Work engagement year 2**	0.031	0.208	0.061	0.097	0.732	1

**Table 3:** Multiple linear regression analysis on exposure to technological advances toward work engagement year 2.

**Variables**	**Model 1**			**Model 2**			**Model 3**			**Model 4**		
	**Coefficient** ^**a**^	**SE**	***P* value**	**Coefficient** ^**a**^	**SE**	***P* value**	**Coefficient** ^**a**^	**SE**	***P* value**	**Coefficient** ^**a**^	**SE**	***P* value**
**Constant**	9.51	0.556	<.001	9.492	0.556	<.001	4.421	1.568	.005	7.123	0.390	<.001
**Exposure to technological advances**	0.243	0.051	<.001	0.246	0.051	<.001	0.891	0.193	<.001	−0.010	0.036	.784
**Age**	0.179	0.006	<.001	0.179	0.006	<.001	0.232	0.016	<.001	0.038	0.005	<.001
**Sex**	0.738	0.169	<.001	0.748	0.170	<.001	0.744	0.170	<.001	−0.190	0.119	.110
**Education level**	0.476	0.062	<.001	0.481	0.062	<.001	0.475	0.062	<.001	0.176	0.044	<.001
**Income category**	0.537	0.054	<.001	0.537	0.054	<.001	0.544	0.054	<.001	0.092	0.038	.015
**Industry characteristics**												
**More likely replaced with automation**				−0.125	0.183	0.492	−0.139	0.183	.448	−0.209	0.128	.102
**Exposure to technological advances × age**							−0.013	0.004	<.001			
**Work engagement year 1**										0.627	0.005	<.001

a Unstandardized coefficient.

**Table 4:** Linear regression analysis of exposure to technological advances toward work engagement year 2 stratified by age group.

**Variable**	**Coefficient** ^**a**^	**SE**	***P* value**
**Analysis within age group 20-39 years**			
**Exposure to technological advances**	0.450	0.106	<.001
**Analysis within age group 40-59 years**			
**Exposure to technological advances**	0.153	0.071	.031
**Analysis within age group 60 years and above**			
**Exposure to technological advances**	0.195	0.099	.049

a Unstandardized coefficient, adjusted for sex, education, income, and industry characteristic.

## 4. Discussion

This study demonstrated an association between exposure to technological advances and high work engagement, and this outcome remained consistent after adjusting for several variables, including personal characteristics, work characteristics, and possible interaction variables. However, this relationship was no longer significant after adjusting for baseline work engagement, suggesting that exposure to technological advances did not contribute to the improvement in work engagement over the year. We also observed that age moderated this relationship, and that younger workers had higher work engagement.

Compared with a study by Molino et al,^13^ which similarly identified a positive association with work engagement, our study measured technology exposure rather than acceptance of technology. Exposure is related to workplace conditions, whereas acceptance is more related to individual perspectives. We examined age as a covariate, whereas Molino et al^13^ defined it as a control variable. Therefore, we were able to examine the role of age in the relation between exposure to technological advances and work engagement. We also used longitudinal data and conducted the study after the COVID-19 pandemic. Sewall et al^23^ have stated that digital technology use during the COVID-19 pandemic did not induce depression, anxiety, or suicidal ideation, questioning the assumption that pandemic-related technology use affected well-being. Therefore, it is possible that technology use and technological advances had a positive effect, as shown in our third model, during and after the pandemic situation.

Although some studies on the role of exposure to technological advances have found an association with negative outcomes such as anxiety, depression, and job insecurity, we found that exposure to technological advances was associated with high work engagement when baseline work engagement was not adjusted for. Our findings therefore indicate that exposure to technological advances has a positive outcome. However, in real workplace practice, if other job resources are available and baseline work engagement is maintained, the association between exposure to technological advances and work engagement may be reduced.

Our findings showed that baseline work engagement status was strongly related to future work engagement. Employees with good work engagement were likely to be better prepared to manage their work demands and allocate resources.^24^ Moreover, they may work more efficiently to achieve their goals; according to the JD-R model, this is an indication of job crafting.^15^ The present findings suggest that good baseline work engagement functions as a good job resource, and contributes toward job crafting and better subsequent work engagement. Thus, exposure to technological advances seems to have less effect on future work engagement, although a positive association was found in Model 3. Therefore, our hypothesis was partly accepted; longitudinal observations showed that exposure to technological advances was not significantly associated with work engagement.

The JD-R model helps to explain why exposure to technological advances has a positive effect on work engagement.^15^ Work engagement is known to increase according to the resources that workers have; these include job resources and personal resources. Technological advances may function as a job resource that helps to make work more efficient.^25^ A reduction in manual work supports workers in performing their tasks more efficiently. Furthermore, workers have more autonomy in completing their work tasks when using technological advances. Therefore, exposure to technological advances functions mainly as a job resource that makes work more efficient.

We also found that the effect of age may modify the association between exposure to technological advances and work engagement. The coefficient of the relationship reduced with increasing age, and we concluded that (older) age had a moderating effect on the association between exposure to technological advances and work engagement. This may explain the recent finding that although the adoption of technology has increased among older people, it is still lower than among younger people.^16^ Younger people may also be more likely to be engaged in jobs that are more strongly affected by technological advances.

Our study included a large number of participants, used longitudinal data, and used data stratified to represent workforce conditions in Japan, one of the countries with a stated high commitment to technological advances.^3^ We believe that our findings reflect current conditions in the Japanese workforce and perhaps conditions in other developed countries. Although most of our participants were from industries less likely to have their jobs automated, exposure to technological advances still played an important role in work engagement if baseline levels of work engagement were not taken into account.

There were several study limitations. First, we did not measure both positive and negative outcomes related to exposure to technological advances. Second, to measure exposure to technological advances, we used a numerical scale that possibly assessed both positive and negative experiences of exposure. Participants with similar scores may have had different experiences, either positive or negative, of exposure to technological advances. Third, we were aware of the risk of attrition bias owing to different characteristics between respondents and nonrespondents; this may have caused overestimation or underestimation when interpreting the results. However, we believe that our data are similar to those from other national surveys, and cover older workers who often work in the informal economy or in contract-based jobs.

We suggest that future studies should simultaneously measure both positive and negative outcomes of exposure to technological advances and include assessment of the experience of such exposure. This type of study is important for generating useful information that could inform national policies on installing new technology in industries.

## Data Availability

The data underlying this article will be shared on reasonable request to the corresponding author.
